# Successful treatment of *MAP2K1* mutant stage IV-M1d melanoma with trametinib plus low-dose dabrafenib: a case report

**DOI:** 10.3389/fmed.2024.1436774

**Published:** 2024-09-09

**Authors:** Iris Dirven, Evan Calliauw, Gil Awada, Manon Vounckx, Jolien I. Kessels, Bart Neyns

**Affiliations:** Department of Medical Oncology, Universitair Ziekenhuis Brussel (UZ Brussel) and Vrije Universiteit Brussel (VUB), Brussels, Belgium

**Keywords:** MAP2K1-mutation, MEK1-mutation, stage IV melanoma, trametinib, MEK-inhibitor, focal post-radiation necrosis, brain metastasis, case report

## Abstract

Clonal MAPK-pathway activating mutations in the *MAP2K1 (MEK1)* gene are present in approximately 9% of cutaneous melanomas. These mutations are divided into three classes: RAF-dependent, RAF-regulated, RAF-independent. Cell lines with class-2 or RAF-regulated *MAP2K1*-mutations are most responsive to MEK-inhibitors. We present a patient with a class-2 *MAP2K1*-mutant stage IV-M1d melanoma who experienced extra- and intracranial progressive disease following treatment with immune-checkpoint inhibitors. The patient was treated with the MEK-inhibitor trametinib (2 mg OD) to which a low-dose of dabrafenib (50 mg BID) was added to mitigate skin-toxicity. Following documentation of a partial response (PR), she developed one new, and increase in volume of two pre-existing brain metastases that were treated with stereotactic radiosurgery (SRS) while continuing trametinib and dabrafenib. Thereafter, a deep partial radiologic and metabolic response both extra-and intra-cranially was achieved and is ongoing 88 weeks after initiating trametinib. She experienced no grade > 2 adverse events. Focal post-radiation necrosis at site of an irradiated brain metastasis developed 9 months after SRS and is successfully being treated with low-dose bevacizumab. This is the first published case of a durable intracranial disease control with the MEK-inhibitor trametinib of a stage IV-M1d class-2 *MAP2K1*-mutant melanoma. This illustrates the utility of NGS profiles that include class-1/2 *MAP2K1*-mutations in patients with melanoma and other malignancies to provide valuable information on a potentially active individualized treatment option. A prospective clinical trial that further evaluates the efficacy of MEK-inhibitor therapies in *MAP2K1*-mutated tumors is justified.

## 1 Introduction

Activating mutations of the mitogen-activated protein kinase (MAPK)-pathway drive proliferation, invasion and metastasis of melanoma (1, 2). Mutations in *BRAF* (mostly V600E/K), *NRAS* (mostly Q61R/K/L) and *NF1* occur in approximately 50, 30, and 25% of cutaneous melanomas, respectively, and are mutually exclusive (3). Patients with triple wild-type melanoma may carry other oncogenic driver mutations (e.g., *KIT*, *MAP2K1*), which mostly cause direct or indirect activation of the MAPK-pathway (4).

Regardless of mutational status, improved overall survival (OS) can be achieved in advanced melanoma with immune checkpoint blockade (ICB) (5, 6). Additionally, targeted therapy with BRAF-/MEK-inhibitors increases survival in *BRAF^V600^*mutant melanoma (7–10). To date, no targeted therapy improves OS in *BRAF^V600^*wild-type melanoma. Preclinical data indicate that MEK-inhibition can be effective in triple wild-type melanoma (11). Arm B of the phase II clinical trial TraMel-WT evaluated trametinib (competitive MEK1/2-inhibitor) combined with low-dose dabrafenib (to mitigate MEK-inhibitor-induced skin-toxicity) in 24 patients with pretreated *BRAF*^V600^/*NRAS^Q61^*wild-type melanoma (NCT04059224). The overall response rate was 29%, median progression-free survival (mPFS) and mOS were 13.3 and 54.3 weeks, respectively (12).

*MAP2K1*, also known as *MAPK/ERK-kinase-1* (*MEK1*), is a serine/threonine/tyrosine kinase that is activated by upstream RAF kinases. Clonal mutations in *MAP2K1* are present in approximately 9% of cutaneous melanomas and activate the MAPK-pathway (4).

In this report, we present in more detail the case of a patient with a triple wild-type, class-2 *MAP2K1*-mutant AJCC stage IV-M1d melanoma, treated in the TraMel-WT trial, who had a deep and durable response to MEK-inhibitor treatment with excellent treatment tolerance (12). The patient provided written informed consent for publication.

## 2 Case description

**Figure 1:**
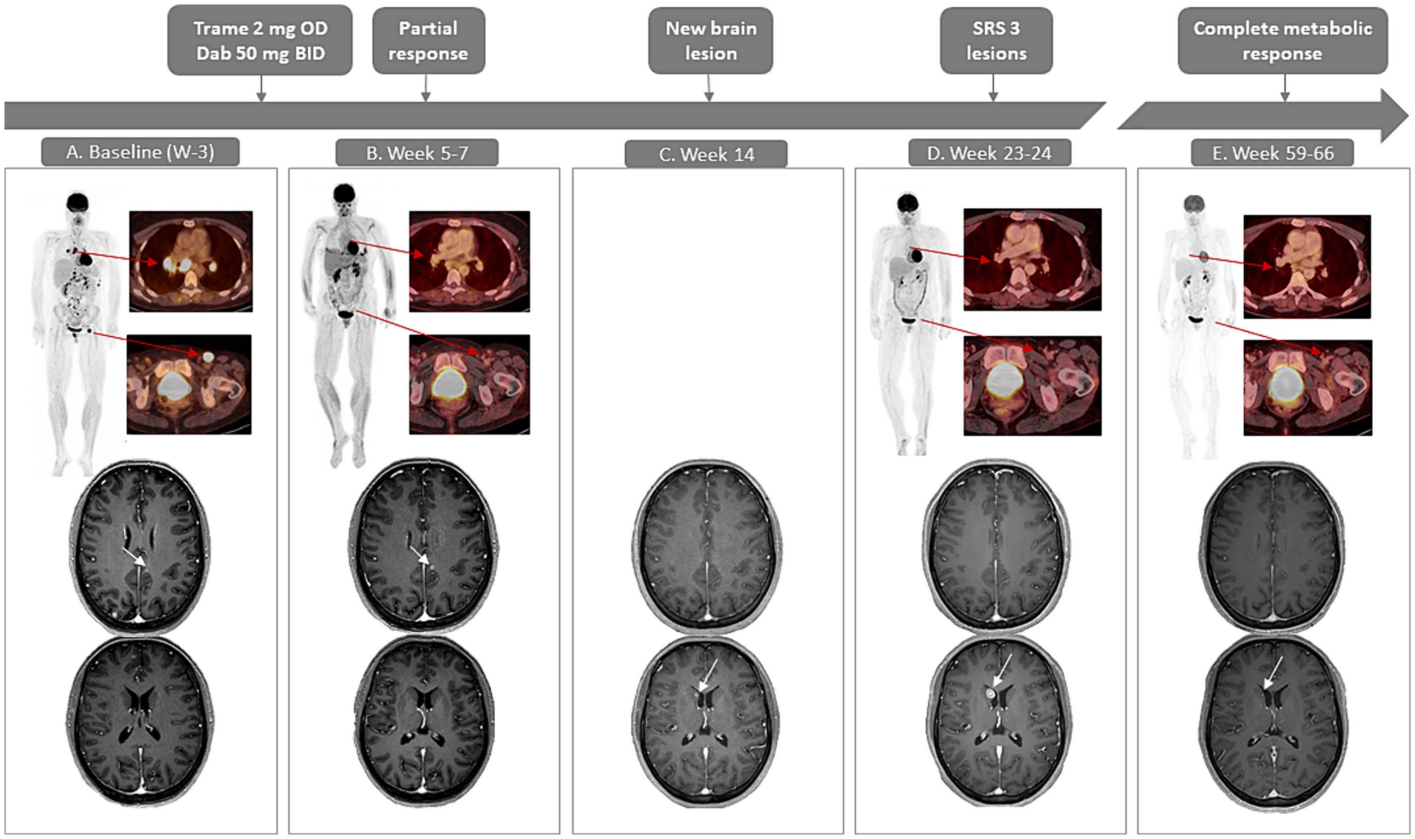
Case presentation with radiological evaluation with [^18^F]FDG-PET/CT (maximum intensity projections and fused axial images) and contrast-enhanced T1-weighted MRI of the brain. **(A)** Baseline, week minus 3 shows hypermetabolic supra-and infradiaphragmatic lesions on PET/CT. In the brain, a lesion can be noted parafalcine in the left (L) occipital lobe (4 mm; upper image) and no lesion is noted in the right (R) nucleus caudatus (lower image). **(B)** PET/CT in week 7 shows a significant decrease in metabolic activity as well as a decrease in size of the lesions (unconfirmed partial response). Brain MRI at week 5 shows a slight increase of the L occipital parafalcine lesion (7 mm, upper image), however there are no new lesions and considering a lead time bias of 3 weeks the treatment is continued. **(C)** On brain MRI in week 14, the parafalcine L occipital lesion has disappeared (upper image), a new lesion in the nucleus caudatus has emerged (lower image). **(D)** PET/CT in week 24 shows a metabolic remission extracranially. MRI shows an increased size of the lesion on the nucleus caudatus (lower image); SRS is performed for this lesion and 2 other progressive lesions (not shown). **(E)** PET-CT in week 66 shows a sustained complete metabolic remission, MRI in week 59 shows a shrinkage of the lesion in the nucleus caudatus (lower image), no other intracranial lesions are detectable on MRI. BID, twice a day; dab, dabrafenib; OD, once daily; SRS, stereotactic radiosurgery; trame, trametinib.

A 40-year-old Caucasian female was diagnosed with a pT4bN1aM0 *BRAF*^V600^wild-type cutaneous nodular melanoma on the left shoulder. During adjuvant pembrolizumab treatment, a solitary subcutaneous metastasis was resected and irradiated. One year after diagnosis, [18F]-fluorodeoxyglucose-positron emission tomography computed tomography ([^18^F]FDG-PET/CT) revealed supra- and infradiaphragmatic lymph node and bone metastases. Treatment with ipilimumab/nivolumab was initiated. She developed immune-related colitis after two cycles and progressed with seven new brain metastases (AJCC stage IV-1Md) (Figure 1A). Comprehensive genomic profiling through next generation sequencing (NGS) revealed a clonal class-2 RAF-regulated *MAP2K1* mutation (Q58_E62del, allele frequency 37%, in-frame-deletion; the complete NGS results can be found in Table 1). The patient initiated trametinib 2 mg once and dabrafenib 50 mg twice daily in the TraMel-WT trial in September 2022. Low-dose dabrafenib was associated upfront to mitigate skin-toxicity (12, 13). She did not receive any local intracranial therapy at this time for the brain metastases. After 5 weeks, brain MRI revealed no new lesions and baseline lesions were considered stable according to RANO-BM criteria (14). Response assessment in week 7 with [^18^F]FDG-PET/CT showed complete metabolic response of extracranial lesions (Figure 1B). After 14 weeks of treatment, brain MRI indicated complete regression of five brain metastases. However, there was one new lesion (6 mm longest diameter) and an increased diameter of two preexisting metastases (Figure 1C). At the patient’s request, close surveillance rather than immediate radiotherapy was applied while continuing trametinib and dabrafenib. Following confirmed progression in these three lesions nearly 2 months later, they were treated with stereotactic radiosurgery (1 × 20 Gy) (Figure 1D). A follow-up MRI (week 32) showed decrease in all three lesions and no new lesions. After 66 weeks of treatment, the patient remained in complete metabolic remission extracranially (Figure 1E). The week-59 brain MRI confirmed a continuing decrease in diameter of one of the irradiated lesions, and complete regression of the others (Figure 1E).

**Table 1:** Comprehensive genomic profiling of the presented patient.

Gene	Shortcode	VAF (%)	Biological class	Clinical class
MAP2K1	Q58_E62del	37	**Likely pathogenic^1^**	Tier IB
RB1	/	41	**Likely pathogenic^1^**	Tier III
LRP1B	W3334*	15	**Likely pathogenic^1^**	Tier III
LRP1B	I2644T	60	VUS	Tier III
LRP1B	D3049E	53	VUS	Tier III
ZNF217	E914_P915delinsDS	47	VUS	Tier III
GNAS	A436D	45	VUS	Tier III
TET1	P119Q	37	VUS	Tier III
CD276	P185S	31	VUS	Tier III
LRP1B	E547Q	30	VUS	Tier III
PLCG2	N798S	28	VUS	Tier III
SPTA1	S818F	28	VUS	Tier III
IL7R	G434D	26	VUS	Tier III
GRM3	G18K	22	VUS	Tier III

1 These mutations are reported as being likely pathogenic following the comprehensive genomic profiling.

VAF, variant allele frequency; VUS, variant of uncertain significance.

Treatment was well tolerated. She intermittently reported low-grade nausea, pruritus, epigastric pain and fatigue, but did not experience skin-toxicity. No dose reductions of trametinib or dabrafenib were required.

**Figure 2:**
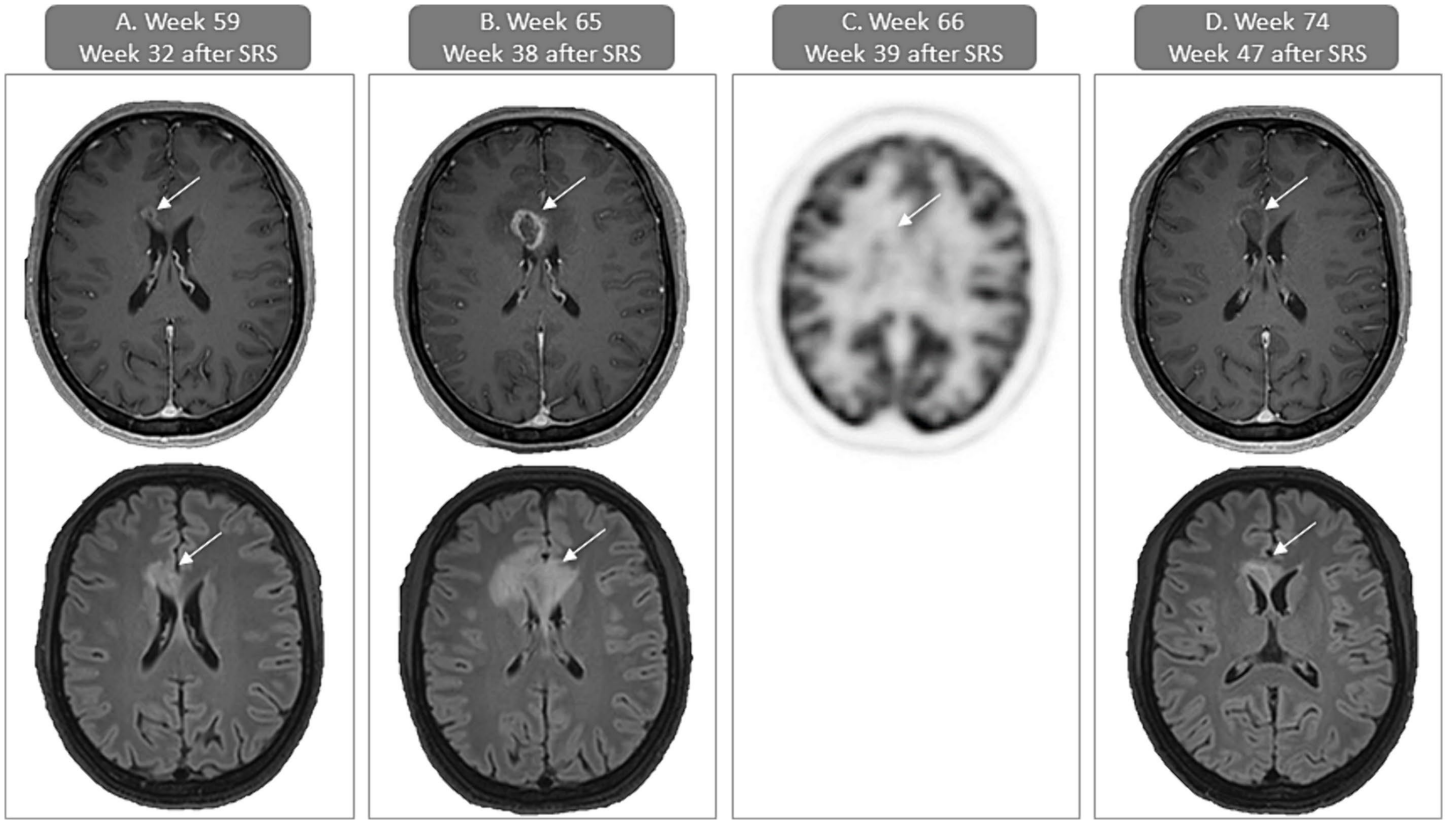
Case presentation of focal post-radiation necrosis of the brain (fRNB) with gadolinium contrast-enhanced T1-weighted and FLAIR MRI of the brain and [^18^F]FDG-PET/CT (FDG-uptake images). **(A)** First sign of fRNB (week 59 or 32 weeks after SRS) with contrast-enhancement in the paramedian frontal right region on the T1-weighted axial image (upper) and surrounding edema on the FLAIR axial image (lower). **(B)** In week 65 or 38 weeks after SRS there is an increase of contrast-enhancement in the paramedian frontal right region on the T1-weighted axial image (upper) and surrounding edema on the FLAIR axial image (lower). **(C)** PET-CT performed 1 week later (week 66 or 39 weeks after SRS) shows no increased uptake of [18F]FDG, supporting the diagnosis of fRNB. **(D)** In week 74 or 8 weeks after bevacizumab initiation there is a decrease of contrast-enhancement in the paramedian frontal right region on the T1-weighted axial image (upper) and surrounding edema on the FLAIR axial image (lower). SRS, stereotactic radiosurgery.

While extra- and intracranial responses persisted, the week-59 brain MRI revealed signs of focal post-radiation necrosis of the brain (fRNB) approximately 8 months after SRS for the lesion in the paramedian frontal right region (Figure 2A). A brain MRI, repeated 6 weeks later, showed increase in contrast-enhancement and size of the region (Figure 2B), which was associated with headaches. In order to differentiate between fRNB and tumor progression, an [^18^F]FDG-PET/CT of the brain was performed and demonstrated focal hypometabolism at the region of the gadolinium-contrast-enhancement, supporting the diagnosis of fRNB. A low-dose bevacizumab treatment regimen (previously established as effective treatment for fRNB) was initiated to treat the perilesional edema (15) (Figure 2C). After two doses of bevacizumab (8 weeks) the headache subsided and MRI showed an important decrease in contrast-enhancement and edema (Figure 2D).

At the moment of writing, after 88 weeks of treatment, the patient continues to have a radiological response and remains on treatment (trametinib with low-dose dabrafenib and bevacizumab), while maintaining an active lifestyle.

## 3 Discussion

No approved therapies have shown to improve OS in patients with *BRAF*^V600^wild-type melanoma who progress on ICB. In melanoma with non-*NRAS*^Q61^/*BRAF*^V600^, MAPK-pathway activating mutations, MEK-inhibitor therapy has shown anti-tumor activity (12, 16, 17). *In vitro* trametinib has activity in triple wild-type melanoma cell lines (18). In *MAP2K1*-mutant melanoma, there is one case report of a partial response to trametinib in a patient with stage IV-M1c melanoma, but resistance developed 3 months later and dose reduction was needed because of trametinib-induced skin-toxicity (Table 2) (19). To the best of our knowledge, this is the first case report of stage IV-M1d *MAP2K1*-mutant melanoma with a durable extracranial complete response and intracranial disease control with trametinib and low-dose dabrafenib. Our patient initially had unconfirmed PR, thereafter oligo-progression in the brain, which was managed locally with SRS while continuing trametinib and dabrafenib. Eventually, a deep and durable disease control, both extra- and intracranially, was achieved.

**Table 2:** Overview of case reports describing patients with an *MAP2K1*-mutation treated with trametinib.

Case	Current case	Krebs et al. (19)	Wang et al. (20)	Cheng et al. (21)	Andritsos et al. (22)	Azorsa et al. (23)	Gounder et al. (24)	Kumamoto et al. (25)	Lorillon et al. (26)	Roeser et al. (27)
Age, gender	40 y, F	55 y, M	52 y, F	67 y, M	52 y, M	16 y, M	62 y, M	18 y, M	18 y, M	65 y, F
Tumor type	Melanoma	Melanoma	Colorectal cancer	Nonsquamous NSCLC	vHCL	LCH	non-LCH*	Non-LCH*	Pulmonary LCH	LCH
AJCC stage	Stage IV-M1d	Stage IV-M1c	Stage IV	Stage IV	/	/	**/**	/	/	/
Prior systemic treatment	Pembrolizumab, ipilimumab/nivo-lumab	Ipilimumab	FOLFOX, FOLFOX + panitumumab	Carboplatin + paclitaxel, pembrolizumab, carboplatin + pemetrexed + bevacizumab, docetaxel	Cladribine, BL22, rituximab/pento-statin, rituximab, ibrutinib, bendamustine/ retuximab, allogeneic transplantation	LCH-III therapy, afuresertib, cytarabine, vincristine, clofarabine	Rituximab/ bendamustine doxorubicin/ cyclophosphamide	Steroids, ICE, cladribine	Cladribine	Vinblastine, cladribine
*MAP2K1* mutation	p.Q58-E62del (Class-2)	p.C121S (Class-2) P124A (Unknown)	p.E102-I103del (Class-3)	p.K57N (Class-2)	p.K57N (Class-2)	p.L98-K104del (Class-3)	p.F53L (Class-2)	p.F53L (Class-2)	p.E102-I103del (Class-3)	p.E102-I103del (Class-3)
Trametinib dosing	Trametinib (2 mg OD) + Dabrafenib (50 mg BID)	2 mg OD - decreased to 1 mg OD - re-challenge 2 mg upon first PD	Not reported	2 mg OD	2 mg OD	2 mg OD	2 mg OD	1st trial: 2 mg OD – decreased to 0.5 mg OD 2nd trial: 2 mg OD	2 mg OD	2 mg OD – decreased to 1.5 mg
Response	Initial unconfirmed PR, followed by PD intracranial; PR after local treatment	PR, then PD	Decline in CA19.9, PD on first radiographic assessment	Mixed response (response in some lesions, PD in others)	Clinical near CR, at 6 months SD with decreased disease activity	PD	CR (>2 years)	1st trial: PD in liver 2nd trial: PR	PR	Initial PR, then PD after 5 months; subsequent CR under cobimetinib
Toxicity	G1 itching G1 nausea G1 epigastric pain G1 fatigue	G3 fatigue G3 skin rash (dose reduction)	Not reported	Acneiform rash, diarrhea, nausea	G1 Acneiform facial rash	Not reported	Not reported	1st trial: G4 hepatotoxicity 2nd trial: G2-3 skin toxicity (treatment discontinuation)	G1 Acneiform skin rash G1 CPK elevation	Cardiac toxicity with decreased LVEF (dose reduction)

*Histiocytic sarcoma. Abbrevations: CPK: creatine phosphokinase; CR: complete response; F: female; G: grade; LCH: Langerhans cell histiocytosis; LVEF: left ventricle ejection fraction; M: male; PD: progressive disease; PR: partial response; NSCLC: non-small-cell lung cancer; SD: stable disease; vHCL: variant hairy cell leukemia; y: years (19–27).

The prognosis and natural evolution of AJCC stage IV-M1d, *BRAF*-wild-type melanoma with active brain metastasis progressing on ICB is very poor. In this patient, the brain metastases were successfully controlled: the majority disappeared with trametinib and no new lesions emerged following SRS, maintaining disease control for more than 20 months at moment of writing.

MAP2K1 mutations can be classified into three groups. Class-1, RAF-dependent *MAP2K1*-mutations are dependent on upstream RAF-activation to induce high levels of activated phosphorylated ERK (pERK), likewise the pathway is self-limited by feedback inhibition of RAS or RAF. Class-2, RAF-regulated *MAP2K1*-mutations are partially dependent on RAF-activation but have varying amounts of RAF-independent activity. Finally, class-3, RAF-independent mutations induce high levels of pERK without upstream RAF-activation and are not susceptible to feedback inhibition (11). Class-1/2 *MAP2K1*-mutations are usually associated with upstream mutations in *RAS*, *RAF*, or *NF1*, while class-3 *MAP2K1*-mutations do not coexist with other mutations. *MAP2K1*-mutations have been successfully targeted *in vitro* with MEK-inhibitors that preferably bind to the inactive, unphosphorylated form of the MEK1-enzyme. Both class-1 and -2 *MAP2K1*-mutations are (partially) RAF-dependent, suggesting a significant inactive fraction of the mutant MEK1-enzymes and thus sensitivity to trametinib. Class-3 mutated proteins on the other hand are resistant to allosteric MEK-inhibitors due to their permanently active conformation (11). The *MAP2K1*-mutation (Q58-E62del) found in our patient, results in an in-frame deletion and is classified as class-2 RAF-regulated *MAP2K1*-mutation (28). In accordance with these preclinical results, the melanoma lesions in our case responded to trametinib.

A recent retrospective review of the AACR genie, a clinico-genomic database showed that co-occurring MAPK-pathway mutations (e.g., *NRAS*, *NF1*) are significantly more likely with class-1 *MAP2K1*-mutations (82.3%) compared to class-2 (30.9%) and class-3 (10.6%) in any tumor type. This highlights that additional activation of the MAPK-pathway is needed to induce malignant cell growth in class-1 RAF-dependent *MAP2K1*-mutant tumors, that this is to a lesser extent necessary in class-2 *MAP2K1*-mutant and not necessary in class-3 *MAP2K1*-mutant tumors. Class-2/3 *MAP2K1*-mutations can therefore act as a driver mutation. Additionally, patients receiving MAPK-inhibitors, with class-2 *MAP2K1*-mutations derived the most benefit, translating to a longer PFS (4.0 months) and duration of response (23.8 months) (29).

Several other cases of successful MEK-inhibitor use in *MAP2K1*-mutant malignancies have been documented, primarily non-Langerhans cell histiocytosis and hairy cell leukemia (Table 2) (30). Responses were observed in cases with class-2 and -3 *MAP2K1-*mutations, however in the class-3 cases, responses were mostly short-lived. These data show the importance of precision oncology and systematic genomic analysis through NGS in both triple wild-type melanoma and other malignancies in which no classical driver mutations have been identified to expand possible treatment options.

Another point of interest is that in five of the seven cases reporting adverse events, rash or acneiform dermatitis were reported (Table 2). In one case this led to treatment discontinuation, in another trametinib dosing was reduced (19, 25). In our patient trametinib-induced skin-toxicity was successfully prevented by adding low-dose BRAF-inhibitor, as previously reported by our group, seemingly without compromising MEK-inhibitor activity (12, 13). Consequently, the patient has an excellent and durable tolerance of full dose trametinib.

Of note, the patient developed fRNB 8 months after SRS, a late side effect of SRS with increasing frequency as more effective therapy for brain metastases becomes available. [18F]FDG-PET/CT helps distinguish fRNB from tumor progression, with fRNB showing decreased [^18^F]FDG uptake (hypometabolism) and tumor progression showing increased uptake (hypermetabolism) (15, 31). Our group recently reported a case series of successful fRNB treatment using low-dose bevacizumab (loading dose of 400 mg, followed by 100 mg q4w) (15). This regimen was effective and well tolerated alongside trametinib and dabrafenib in our patient.

## 4 Conclusion

We report the first case of durable intra- and extracranial response to trametinib, following local control with SRS of intracranial oligo-progression, in a patient with stage IV-M1d class-2 *MAP2K1*-mutant melanoma. Association of low-dose BRAF-inhibitor prevented MEK-inhibitor-induced skin-toxicity. Precision oncology using NGS data to screen for *MAP2K1*-mutations offers valuable treatment insights. A cross-tumor prospective trial is needed to evaluate the efficacy of MEK-inhibitors in *MAP2K1*-mutated tumors.

## Data Availability

The data analyzed in this study is subject to the following licenses/restrictions: the dataset generated for this case report contains confidential patient information but can be made available upon request to interested researchers. Requests to access these datasets should be directed to ID, iris.dirven@uzbrussel.be.
